# Clinical Perspectives of Theranostics

**DOI:** 10.3390/molecules26082232

**Published:** 2021-04-13

**Authors:** Shozo Okamoto, Tohru Shiga, Nagara Tamaki

**Affiliations:** 1 Department of Radiology, Obihiro-Kosei General Hospital, Obihiro 080-0024, Japan; shozo@med.hokudai.ac.jp; 2 Department of Diagnostic Radiology, Hokkaido University Graduate School of Medicine, Sapporo 060-8638, Japan; 3 Advanced Clinical Research Center, Fukushima Global Medical Science Center, Fukushima 960-1295, Japan; tshiga@fmu.ac.jp; 4 Department of Radiology, Kyoto Prefectural University of Medicine, Kyoto 602-8566, Japan

**Keywords:** PET, theranostics, radionuclide therapy, cancer, prognosis

## Abstract

Theranostics is a precision medicine which integrates diagnostic nuclear medicine and radionuclide therapy for various cancers throughout body using suitable tracers and treatment that target specific biological pathways or receptors. This review covers traditional theranostics for thyroid cancer and pheochromocytoma with radioiodine compounds. In addition, recent theranostics of radioimmunotherapy for non-Hodgkin lymphoma, and treatment of bone metastasis using bone seeking radiopharmaceuticals are described. Furthermore, new radiopharmaceuticals for prostatic cancer and pancreatic cancer have been added. Of particular, F-18 Fluoro-2-Deoxyglucose (FDG) Positron Emission Tomography (PET) is often used for treatment monitoring and estimating patient outcome. A recent clinical study highlighted the ability of alpha-radiotherapy with high linear energy transfer (LET) to overcome treatment resistance to beta--particle therapy. Theranostics will become an ever-increasing part of clinical nuclear medicine.

## 1. Introduction

Theranostics is a re-emerging new medical term of combination of diagnostic and therapeutic techniques using suitable drug combination [1]. The aim of theranostics is to provide personalized medicine to patients with cancer by suitable radionuclide imaging and radiotherapeutics which targets specific biological pathways and receptors.

Theranostics has long been applied to treat thyroid cancer and neuroblastomas using suitable radiolabeled compounds, I-131 iodine and I-131-iodine-meta-iodobenzylguanidine (MIBG). More recently, theranostics has been refocused with the advent of new therapeutic antibodies and small molecules which can be transformed into theranostic agents through radio-conjugating with radioactive isotope materials [2]. A number of PET compounds labeled with Ga-68 or F-18 have been used for imaging, while beta- or alfa-emitting sister compounds are applied for therapy [3,4,5].

Theranostics plays an important role for both detection of malignant lesions throughout the body using tumor affinity compounds, and also treating lesions with radiotherapy emitted from beta- or alfa-emission from the same targeted radiolabeled compounds. Table 1 summarizes various combinations of diagnostic imaging and internal therapy with radiopharmaceuticals for the same target molecule. Thus, systematic imaging and radioisotope therapy is permitted with the use of suitable radiolabeled compounds. There are a number of reports indicating not only the diagnostic values but also better prognostic values of oncology therapy as compared to the conventional systemic chemotherapy [6,7].

This is important to find a suitable cell target for specific radionuclide imaging in combination with target radiotherapy. The first three fields cover specific targets accumulated in the cancer cells (internalization) using suitable radiolabeled agents. Radiolabeled iodine is accumulated in thyroid cancer. Radiolabeled MIBG goes into cancer cells in malignant pheochromocytoma and paraganglioma. Radiolabeled somatostatin receptor is trapped in neuroendocrine tumors (Table 1). On the other hand, radiolabeled agents do not go into the cancer cells in non-Hodgkin lymphoma or bone metastasis (not internalization). Instead, these agents accumulate near cancer cells with specific targets, such as CD20 antigen on the surface of tumor cell membranes in lymphoma and bone reaction near tumor cells in osteoblastic bone metastases (Table 1).

## 2. Prognostic Value of Theranostics for Thyroid Cancer

Radioiodine therapy with I-131 iodine is a traditional and well-established treatment modality for adjuvant postsurgical ablation of thyroid remnant tissue and therapy of nonresectable local recurrences, lymph node, and distant metastases [8,9,10].

**Figure 1:**
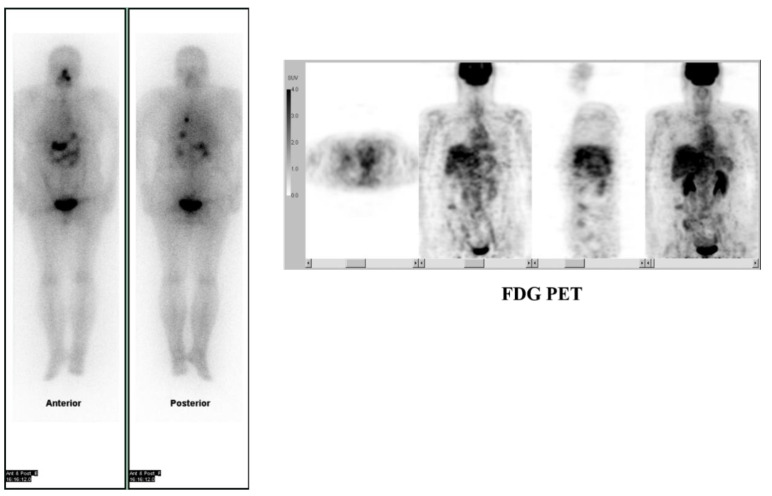
A case with thyroid cancer showing positive I-131 and negative FDG uptake in the lung.

**Figure 2:**
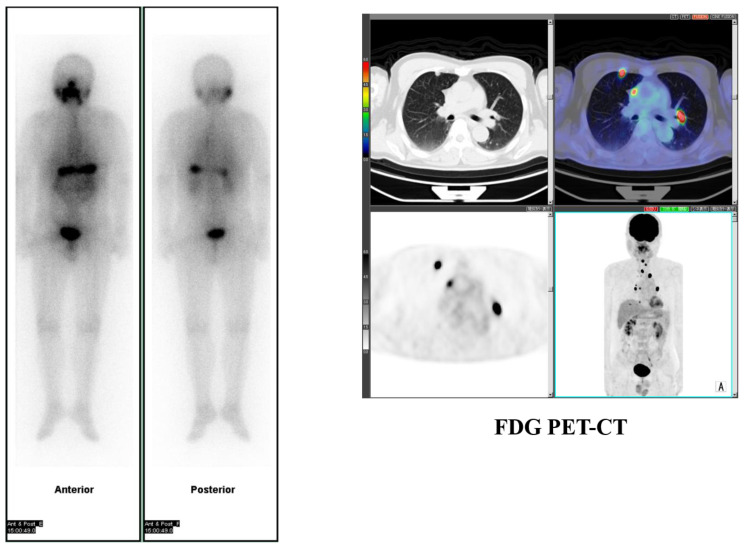
A case with thyroid cancer showing negative I-131 and positive FDG uptake in the lung.

Differentiated thyroid cancer has strong affinity to I-131 and thus, shows good response to iodine treatment. Dedifferentiated thyroid cancer lesions, on the other hand, tend to lose radioiodine affinity, but instead show an increased glucose metabolism [20]. Both histological and imaging studies provide evidence that increased glucose uptake correlates with a higher grade of malignancy and a worse prognosis [21,22,23,24]. Accordingly, FDG PET is considered a valuable tool for the detection of radioiodine-negative metastases in the follow-up of patients with DTC [25,26,27,28] (Figure 1 and Figure 2). In addition, FDG-PET holds a promise as a valuable tool for predicting long prognosis of thyroid cancer.

We have analyzed the predictive value of FDG-PET in comparison with radioiodine uptake in 141 high-risk patients with differentiated thyroid cancer with radioiodine therapy after total thyroidectomy [29]. Our results indicate that FDG PET was more predictive for long-term survival, whereas radioiodine uptake was more important for short-term response. Therefore, FDG PET after thyroid remnant ablation should hold a prognostic value for management of high-risk patients with differentiated thyroid cancer [29].

## 3. Prognostic Value of Theranostics for Neuroendocrine Tumor

Pheochromocytoma and paraganglioma may develop metastatic lesions. The mean expected survival time with pheochromocytoma is 20.7 years and with paraganglioma is 9.8 years [11,30]. After surgery, systemic chemotherapy is commonly performed for treating remaining and metastatic lesions; however, the survival benefits are not clear because of the lack of prospective comparative studies [31,32,33].

Iodine-131-meta-iodobenzylguanidine (I-131 MIBG) is a substrate of the norepinephrine transporter system and the structure is similar to norepinephrine [34]. Norepinephrine works as a neurotransmitter in the central and autonomic nervous systems. The concentrations of norepinephrine in the nervous systems are regulated by the norepinephrine transporter. I-131 MIBG also accumulates; however, unlike norepinephrine, I-131 MIBG has little or no affinity for adrenergic receptors [35]. I-131 MIBG radiotherapy has been used to treat neuroendocrine tumors (NETs), including metastatic pheochromocytoma, paraganglioma, neuroblastoma, medullary thyroid carcinoma, and carcinoid tumors that have the uptake-1 mechanism [12,34,36].

I-131 MIBG radiotherapy has shown some survival benefits in metastatic NETs. The European Association of Nuclear Medicine (EANM) clinical guidelines for I-131 MIBG radiotherapy suggest a repeated treatment protocol, although none currently exists [37]. The existing single-high-dose I-131 MIBG radiotherapy (444 MBq/kg) has been shown to have some benefits for patients with metastatic NETs. However, this protocol increases adverse effects and it requires alternative therapeutic approaches.

**Figure 3:**
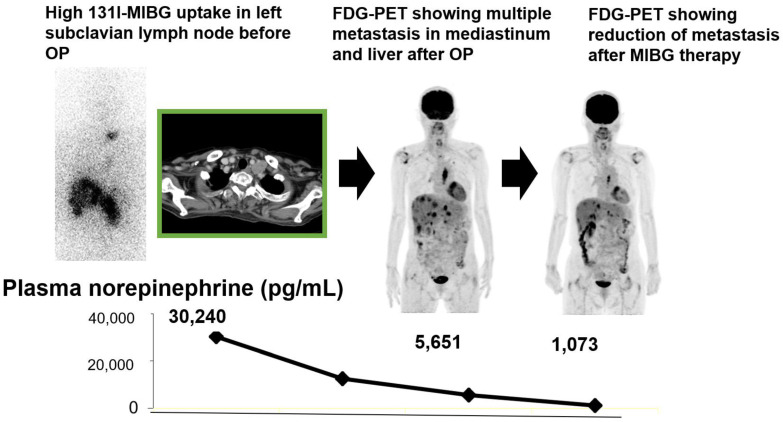
A case with pheochromocytoma.

A previous study reported that FDG PET maximum standardized uptake value (SUVmax) was reduced after I-131 MIBG radiotherapy in patients considered to have responded, suggesting the value of FDG PET imaging for treatment evaluation in patients with PPGL after I-131 MIBG radiotherapy [38] (Figure 3).

Accordingly, we have evaluated the effects of repeated I-131 MIBG therapy on tumor size and tumor metabolic response in patients with metastatic NETs to find reduced tumor size and tumor metabolic activities according to lesion-based analysis in about one-third of patients and stabilized a majority with this therapy [39]. Thus, we concluded that this relatively short-term repeated I-131 MIBG treatment may have potential as one option in a therapeutic protocol for metastatic NETs [39].

Theranostics has recently been applied for NETs with somatostatin receptor targeting small molecule tracers such as Ga-68 DOTATATE PET, In-111 Pentetreotide, and Peptide Receptor Radionuclide Therapy (PRRT) with Lu-177 DOTATATE therapy. The patients with positive uptake in Ga-68 DOTATATE PET or In-111 Pentetreotide are indication for Lu-177 DOTATATE treatment in the European Medicine Agency (EMA) and Food and Drug Administration (FDA). Progression free survival (PFS) rate at 20 months was significantly increased from 11% for patients in the control group to 65% in the Lu-177 DOTA-TATE group (*p* < 0.0001) [13].

## 4. Prognostic Value of Radioimmunotherapy of Non-Hodgkin Lymphoma

Non-Hodgkin lymphoma is a common malignancy where I-131 compound has been used for theranostics. Initially 131-iodine-tositumomab was used for imaging and Y-90-ibritumomab-tiuxetan (Zevalin) was used for therapy [14]. However, these initial theranostic trials have failed to become incorporated into mainstream oncologist practice. More recently, lymphoma is effectively treated with 131-iodine-anti-CD20 radioimmunotherapy (RIT) in relapsed/refractory disease following traditional R-CHOP chemotherapy [40,41]. There was no significant toxicity, and also this single-shot theranostic study seems to be superior to all standard therapies. The recent substitution of Lu-177 rituximab, in place of 131-iodine radiolabelling, will facilitate outpatient therapy by minimizing radiation exposure of medical staffs, while retaining the ability to perform individual prospective dosimetry [42]. A recent clinical study using Lu-177 lilotomab satetraxan anti-CD37 monoclonal antibody RIT suggested that tumor radiation absorbed doses comparable with those achieved with 131-iodine-tositumomab anti-CD20 Mab were attained, at red marrow doses below the 2 Gy threshold for myelotoxicity [43]. 

## 5. Prognostic Value of Theranostics for Bone Metastasis

The skeleton is the most common metastatic site in patients with advanced cancer, particularly in prostatic cancer. Pain is a major healthcare problem in patients with bone metastases. Bone-seeking radionuclides indicating an increase in bone turnover that selectively accumulate in the bone are used to treat cancer-induced bone pain. The goals of these guidelines are to assist nuclear medicine practitioners in evaluating patients who might be candidates for radionuclide treatment of bone metastases using beta-emitting radionuclides such as strontium-89 and Samarium-153 ethylenediaminetetramethylenephosphonic acid (EDTMP) [15]. Strontium-89 is an element that behaves biologically like calcium. The accumulation of strontium is identical to that of Tc-99m MDP (methylene diphosphonate). The distribution of Samarium-153 EDTMP is also identical to Tc-99m MDP [16,44].

On the other hand, theranostic approaches for osseous metastasis have long been focused for alpha therapy using Ra-223 for various solid tumors, such as thyroid cancer and prostatic cancer. It is important that radionuclide therapy has not only a palliative effect but also a potential for prolonging progression-free and overall survival [17,45,46]. This important randomized control study nicely showed 3.6 months longer survival by Ra-223 therapy for bone metastasis from castrate-resistant prostatic cancer [17]. Accordingly, the Japanese government has approved insurance coverage for Ra-223 therapy for those with multiple bone metastasis from prostatic cancer [47].

## 6. Alpha vs. Beta-Emitting Isotope Radiochemistry

As previously described, theranostics has been applied by using suitable therapeutic radioisotopes, including alpha- and beta-emission ones over the last century. Beta-emitting radioisotopes have the longest particle pathlength (≤12 mm) and lowest linear energy transfer (LET) (~0.2 keV/μm), supporting the effectiveness in medium to large tumors. Long beta-particle range is advantageous in evenly distributing a radiation dose in heterogeneous tumors. It can also, though, result in the irradiation of healthy tissue surrounding the tumor site causing a relatively large side effect. Alpha-particles have a moderate pathlength (50–100 μm) and high LET (80 keV/μm) that render them especially suitable for small neoplasms or micro-metastases with such smaller side effects in the surrounding normal organ. A recent clinical study highlighted the ability of alpha-radiotherapy to overcome treatment resistance to beta-particle therapy, prompting a paradigm shift in the approach toward radionuclide therapy [19,48].

## 7. Future Perspectives of Theranostics

More recently, a number of new radio-labeled pharmaceutical compounds have been investigated for promoting theranosticis. One of the most exciting new theranostic agents is the prostate specific membrane antigen (PSMA) binding compound for prostatic cancer developed at Heidelberg University and in many researches worldwide [49]. Prostatic cancer is the second most common cancer in men worldwide with an estimated about a million 1,276,000 new patients. About 30% of men experience biochemical recurrence, often progress to castration resistant prostate cancer (CRPC), and there were 359,000 deaths in 2018 [50,51].

**Figure 4:**
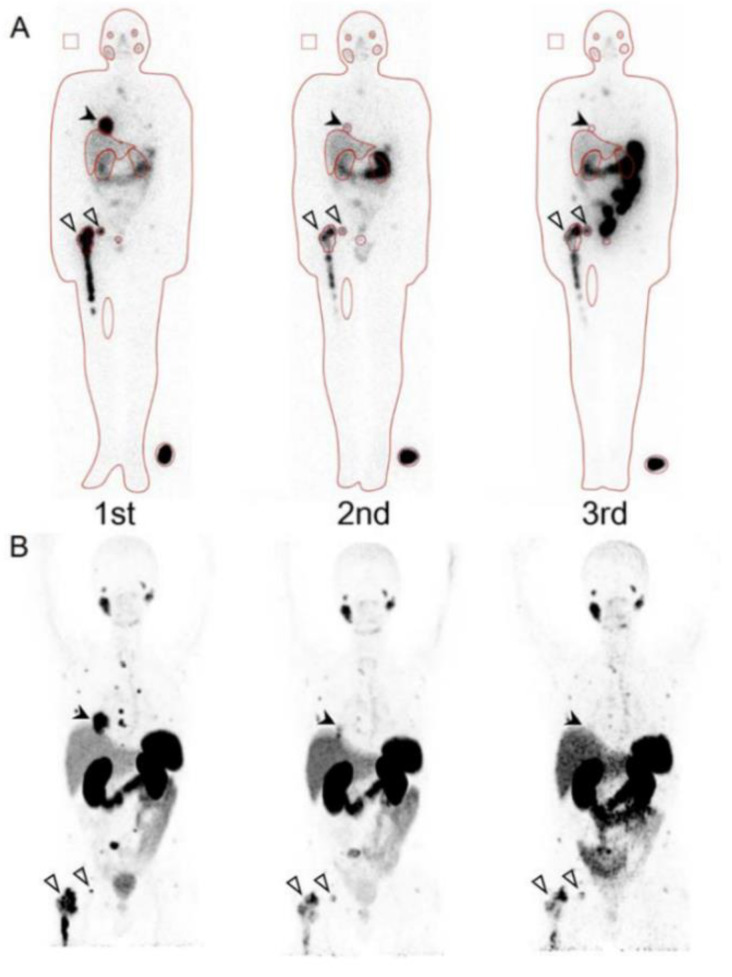
A patient with multiple metastases in lung (black arrow) and bone (white arrows): (**A**): Posttherapeutic Lu-177 prostate specific membrane antigen (PSMA) scintigraphy; (**B**): Pretherapeutic Ga -68 PSMA PET. Copyright 2017, with permission from J. Nucl. Med. [56].

New therapeutic agents such as abiraterone, enzalutamide, and cabazitaxel have been developed in the last decade [52]. On the other hand, diagnostic tools with radionuclide such as Ga-68 or F-18 labelled PSMA have been used for whole body PET imaging to identify metastatic lesions [53,54,55,56]. In addition, Lu-177 or Ac-225 labeled PSMA therapies are expected to have a therapeutic effect by a unique mechanism using radiopharmaceuticals [18,19,57,58]. Lu-177 or Ac-225 PSMA therapies are the main strategies in theranostics at present. PSMA PET detects recurrent and metastatic tumors more accurately than conventional imaging diagnostic tools such as computed tomography (CT), bone scintigraphy (BS), or F-18 Fluciclovine PET [53,54,55,56]. In the study of our group at the Technical University of Munich, the accuracy of Ga-68 PSMA PET was 99.8% which was significantly higher than BS (84.3%) and BS + Single Photon Emission Computed Tomography (SPECT). In another international trial of Ga-68 PSMA-PET in biochemical recurrence, 75% of patients had a positive lesion detected, and both the positive predictive value and sensitivity were 92% [56]. The FDA approved Ga-68 PSMA-PET for patients with suspected prostate cancer metastasis and with suspected prostate cancer recurrence based on elevated serum prostate-specific antigen (PSA) levels in December 2020. Our group also reported that similar uptake is shown between pretherapeutic Ga-68 PSMA-PET and Lu-177 PSMA therapy; therefore, Ga-68 PSMA PET avid lesions are expected the effect of Lu-177 PSMA therapy (Figure 4) [57]. Up to 4 cycles treatments with 6 weeks interval are a standard course of Lu-177 PSMA therapy considered for a kidney absorbed dose at a risk organ.

The remarkable therapeutic effect of Lu-177 PSMA therapy for CRPC has been described in several reports. PSA decline from baseline was seen in 68% of patients [59], greater than or equal to 50% seen in 44–64% of patients [18,58,59,60,61,62]. At least 80% decline was seen in 44% of patients, and 16% achieved an at least 98% PSA decline [58]. Regarding toxicity, there were no treatment-related deaths, and grade 2 or less xerostomia was reported in 8–66% of patients. Grade 1–2 nausea was seen in 6–48%, respectively. Grade 3–4 toxicity was primarily hematologic, including leukopenia (3–32%), thrombocytopenia (5–10%), and anemia (10%). Grade 1–2 renal injury occurred in 10% of patients [58,59]. Ac-225 PSMA therapy is expecting excellent therapeutic effect even for PC resistant to Lu-177 PSMA therapy [63]. PSA decline of more than 50% from baseline was observed in 21–63% of the patients [63,64,65]. This rate is similar to the biochemical response rates of Lu-177 PSMA therapy; however, note that many patients who underwent Ac-225 PSMA therapy were refractory to previous Lu-177 PSMA therapy.

Another exciting theranostic trial is in applying for cancer with poor prognosis. Most theranostic radiopharmaceuticals have so far been applied for cancer with relatively good prognosis. On the other hand, pancreatic adenocarcinoma has an extremely poor prognosis. Five years survival rate of metastatic pancreatic adenocarcinoma is less than 5% [66]. At the time of diagnosis, most patients are ineligible for surgery due to metastatic spread or local invasion. For metastatic disease, cytotoxic chemotherapy is the only treatment choice on most occasions [67]. Neurotensin receptor 1 is highly expressed in ductal pancreatic adenocarcinoma as well as hepatic metastasis but not in normal pancreatic tissue or chronic pancreatitis [67,68]. 3BP-227 is a DOTA-conjugated neurotensin receptor 1 antagonist and Lu-177 labeled 3BP-227 significantly inhibited tumor growth [68]. The first clinical trial of Lu-177 labeled 3BP-227 indicated feasibility of treatment of ductal pancreatic adenocarcinoma [69]. More clinical experiences are warranted to see whether this new agent may prolong survival of this disease.

In external-radiation therapy, the radiation treatment plan is designed to focus the dose on the malignant tumor and to minimize damage to the surrounding tissues. On the other hand, in conventional radionuclide therapy, doses are administered empirically, and there are no guidelines for dosimetry and no personalization. In radionuclide theranostics, dosimetry can be measured in the following two ways: one is to estimate the dose from the kinetic analysis of companion diagnostics, and the other is to measure the dose of the first treatment. The AHASA (as high dose as safety administrated) approach is proposed for radionuclide therapy [70]. Evidence on the relationship between dose and treatment or side effect is now gradually accumulating [71,72,73,74]. A study of theranostics for neuroendocrine tumors reported that dose escalation based on organ risk improved outcome [74]. In this study, they conducted a prospective observational study of 200 patients with advanced metastatic NETs. Patients received additional cycles of treatment until the cumulative renal dose reached 23 Gy or was discontinued due to toxicity. Overall survival was significantly longer when treatment was given until the cumulative renal dose reached 23 Gy. Once the evidence is established, a personalized, dosimetry-based treatment planning will become the mainstream for the purpose of more effective treatment and avoidance of serious side effects.

As in many other radionuclide studies, the injected drug dose is quite small with minimal side effects. On the other hand, a careful attention should be used for suitable radiation dose to each patient. A rapid grow of theranostics for diagnosis and treatment combination has increased a need for nuclear medicine practitioners with suitable training in the field of new nuclear medicine and radionuclide therapy.

## 8. Conclusions

Theranostics is an elegant technique using suitable pairs of radionuclide imaging and radiotherapeutics which targets specific biological pathways and receptors.

This review has outlined old as well as recently developed nuclear theranostics which have entered the clinic as well as evolving imaging and therapy. In particular, new trends toward patient outcome have been focused upon. Theranostics will become an ever-increasing part of clinical nuclear medicine. This will lead to adaptation of personalized molecular targeted radionuclide therapy as a mainstream oncology practice throughout world.

## Figures and Tables

**Table 1:** Combination of diagnostic imaging and radionuclide with radiopharmaceuticals for the same target disease.

Target Disease	Target	Companion Diagnostics for Theranostics	Radionuclide Therapy
Differentiated thyroid cancer	Iodine affinity for hormone synthesis	I-131	I-131 [8,9,10]
Malignant pheochromocytoma, Paraganglioma	Norepinephrine transporter	I-123 MIBG I-131 MIBG	I-131 MIBG [11,12]
Neuroendocrine tumor	Somatostatine receptor	In-111 Pentetreotide Ga-68 DOTATE Ga-68 DOTATOC	Lu-177 DOTATATE [13] Lu-177 DOTATOC Y-90 DOTATATE Y-90 DOTATOC
B-cell non-Hodgkin malignant lymphoma	Anti-CD20 receptor	In-111 Ibritumomab Tiuxetan I-131 rituximab	Y-90 Ibritumomab Tiuxetan [14] Lu-177 rituximab
Bone metastasis	Bone turnover	Tc-99m bone scan	Sr-89 [15,16] Sm-153 EDTMP [16] Ra-223 [17]
Castration-resistant prostatic cancer	Prostate specific membrane antigen	Ga-68 PSMA F-18 PSMA	Lu-177 PSMA [18] Ac-225 PSMA [19]

## Data Availability

No new data were created or analyzed in this study. Data sharing is not applicable to this article.
